# Orthologs of human circulating miRNAs associated with hepatocellular carcinoma are elevated in mouse plasma months before tumour detection

**DOI:** 10.1038/s41598-022-15061-5

**Published:** 2022-06-28

**Authors:** Liang-Hao Ding, Christina M. Fallgren, Yongjia Yu, Maureen McCarthy, Elijah F. Edmondson, Robert L. Ullrich, Michael. M. Weil, Michael D. Story

**Affiliations:** 1 Department of Radiation Oncology, The University of Texas Southwestern Medical Center, Dallas, TX 75390 USA; 2 Department of Environmental & Radiological Health Sciences, Colorado State University, Fort Collins, CO 80523 USA; 3 Department of Radiation Oncology, The University of Texas Medical Branch, Galveston, TX 77555 USA; 4 NASA Johnson Space Center, Houston, TX 77058 USA; 5 Frederick National Laboratory for Cancer Research, Frederick, MD 21702 USA; 6 Radiation Effects Research Foundation, Hiroshima City, Japan; 7 Harold C. Simmons Comprehensive Cancer Center, The University of Texas Southwestern Medical Center, Dallas, TX 75390 USA

**Keywords:** Liver cancer, Tumour biomarkers

## Abstract

Research examining the potential for circulating miRNA to serve as markers for preneoplastic lesions or early-stage hepatocellular carcinoma (HCC) is hindered by the difficulties of obtaining samples from asymptomatic individuals. As a surrogate for human samples, we identified hub miRNAs in gene co-expression networks using HCC-bearing C3H mice. We confirmed 38 hub miRNAs as associated with HCC in F2 hybrid mice derived from radiogenic HCC susceptible and resistant founders. When compared to a panel of 12 circulating miRNAs associated with human HCC, two had no mouse ortholog and 7 of the remaining 10 miRNAs overlapped with the 38 mouse HCC hub miRNAs. Using small RNA sequencing data generated from serially collected plasma samples in F2 mice, we examined the temporal levels of these 7 circulating miRNAs and found that the levels of 4 human circulating markers, miR-122-5p, miR-100-5p, miR-34a-5p and miR-365-3p increased linearly as the time approaching HCC detection neared, suggesting a correlation of miRNA levels with oncogenic progression. Estimation of change points in the kinetics of the 4 circulating miRNAs suggested the changes started 17.5 to 6.8 months prior to HCC detection. These data establish these 4 circulating miRNAs as potential sentinels for preneoplastic lesions or early-stage HCC.

## Introduction

Cell-free circulating miRNAs in plasma or serum are associated with various disease conditions including cancer^1–7^. Circulating miRNAs are resistant to RNase degradation because they are either encapsulated with membranous vesicles, such as exosomes, or conjugated with protective protein complexes. They can be obtained by minimally invasive methods, such as peripheral venipuncture followed by standardized centrifugation to separate plasma. These features bring potential clinical values to circulating miRNAs as diagnostic, prognostic, and therapeutic biomarkers for cancer. Several circulating miRNAs were reported to be dysregulated at very early stage of cancers, including Stage I or II of non-small cell lung cancer^8–10^, breast cancer^11^ and glioma^12^. It is suggested that circulating miRNAs could be used for screening of early stage or incipient cancers before clinical symptoms or clear evidence from biopsy or image examination are performed^13^.

Liver cancer is the fourth leading cause of cancer-related death worldwide. It is estimated that, by 2025, > 1 million individuals will be affected by liver cancer annually^14^. Hepatocellular carcinoma (HCC) accounts for ~ 90% of liver cancers. Disease prognosis for HCC is largely driven by tumor stage at the time of diagnosis, with a 5-year survival exceeding 70% for early-stage HCC compared to a median survival of ~ 1–1.5 years for symptomatic advanced-stage cases^15,16^. Altered plasma or serum levels of miRNAs have been well-studied in HCC patients and groups of circulating miRNAs associated with HCC have been identified^17^. The question remains as to whether the changes of the HCC-associated miRNAs are detectable very early or at precancerous stages of cancer and whether they can be candidate markers for early screening.

It is often difficult to obtain human samples from early-stage asymptomatic patients and hence animal models, such as mice, are widely used for studying early biomarkers. Yet, the challenge of using mouse models is to translate the findings to human disease. We hypothesize that the functionally important miRNAs that act as key regulators in signaling pathways are conserved across different species, hence studying these key miRNAs in mice will shed light on human biomarker research.

Weighted co-expression network analysis (WGCNA) constructs gene networks, also called modules, which contain collections of genes with similar expression profiles. The theory behind the analysis is that the genes with closely functional linkages or involved in the same pathway are co-expressed in a synchronized pattern^18,19^. Within a module, genes with the greatest connectivity are identified as hub genes, suggesting important roles within the module. WGCNA also analyzes the association between modules and sample traits, which provides an effective way to explore the mechanisms behind certain traits. The analysis has been widely used in high throughput genomics data to identify key regulators and pathways in animal models as well as human disease^20–24^. In this study, we performed WGCNA analysis using an integrated dataset from gene expression and circulating miRNA profiles in a radiation-induced mouse HCC model. The hub miRNAs identified as associated with HCC were compared with human miRNA markers and then validated in an irradiated mouse model from a genetically heterogeneous stock. The HCC-associated circulating miRNAs were quantified in a time series study to investigate how early the overrepresented miRNAs can be quantified.

## Results

### Construction of gene co-expression networks and identification of HCC-associated modules in mice

**Figure 1:**
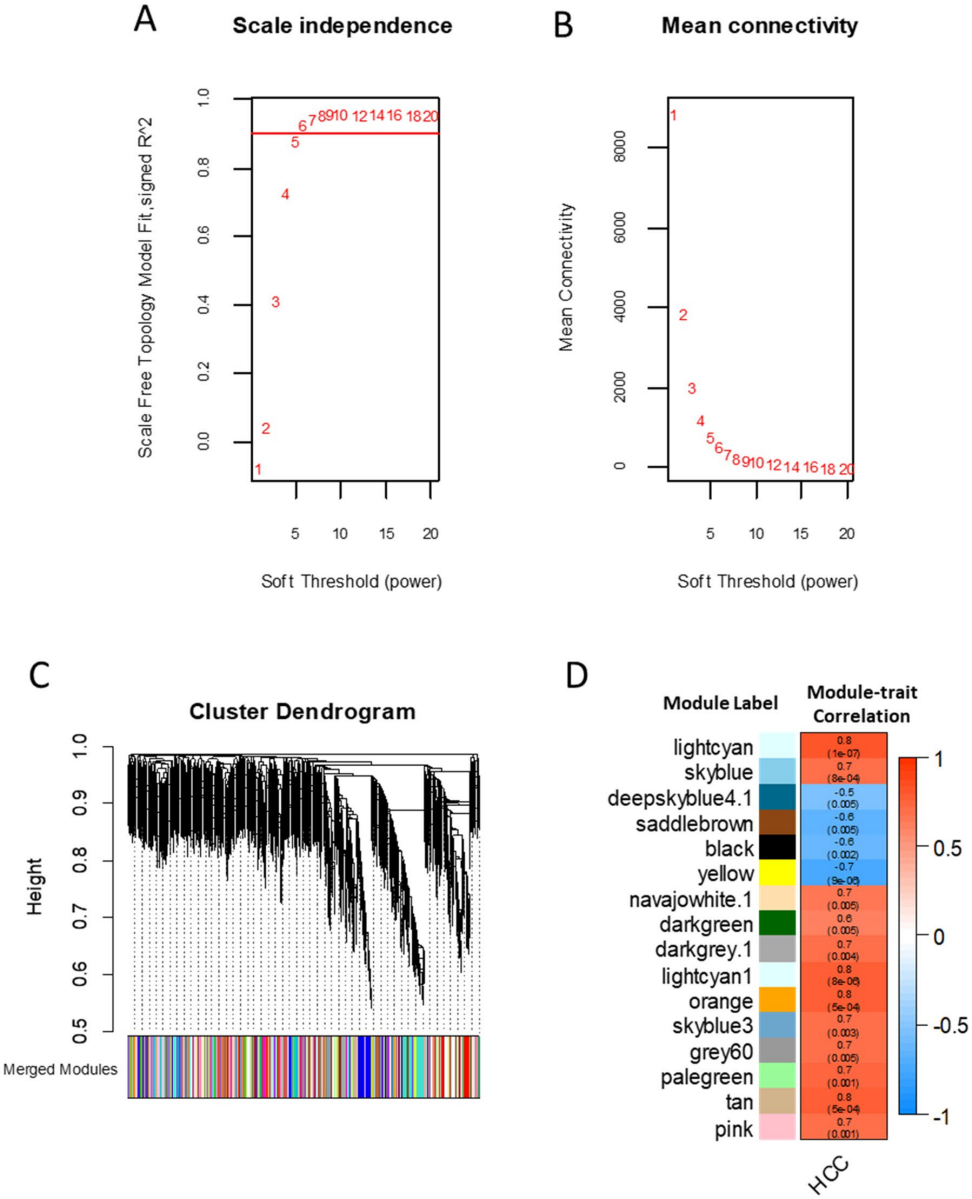
Construction of co-expression networks and identification of HCC-associated modules in C3H mice. (**A**) Relationship of soft threshold and scale-free topology index. We selected 6 as soft thresholding power, which reached R^2^ = 0.93. (**B**) Soft thresholding power as a function of connectivity. (**C**) Clustering dendrogram of genes/miRNA and assigned colors of co-expression modules. Total number of co-expression modules: 758; miRNA-containing modules: 318. (**D**) miRNA containing modules significantly associated with HCC. Numbers displayed in each module: top, correlation coefficient with HCC; bottom, p-value. Colors represent the correlation coefficients between the modules and the HCC phenotype.

We previously published gene expression analysis of HCC in C3H mice^25^. Using the plasma samples from the same cohort we performed small RNA sequencing. Gene expression and circulating miRNA profiles were filtered to remove genes/miRNAs that had small variances. In total, 28,630 gene probes and 1809 circulating miRNAs from each mouse were combined to construct weighted gene co-expression networks (WGCNA). We followed the guideline from the WGCNA package to use soft-thresholding power for calculating an adjacency matrix. By analyzing the network topology we chose a soft-thresholding power as β = 6, which ensured a scale-free network (R^2^ = 0.93) with good connectivity (Fig. 1A,B). A large number of co-expressed modules (758) were constructed from the pooled mRNA and circulating miRNA profiles (Fig. 1C). However, only 16 co-expression modules contained circulating miRNAs and were significantly changed in HCC mice when compared with the non-HCC mice (Fig. 1D).

### Identification of functionally significant hub circulating miRNAs in HCC-associated co-expression modules

**Figure 2:**
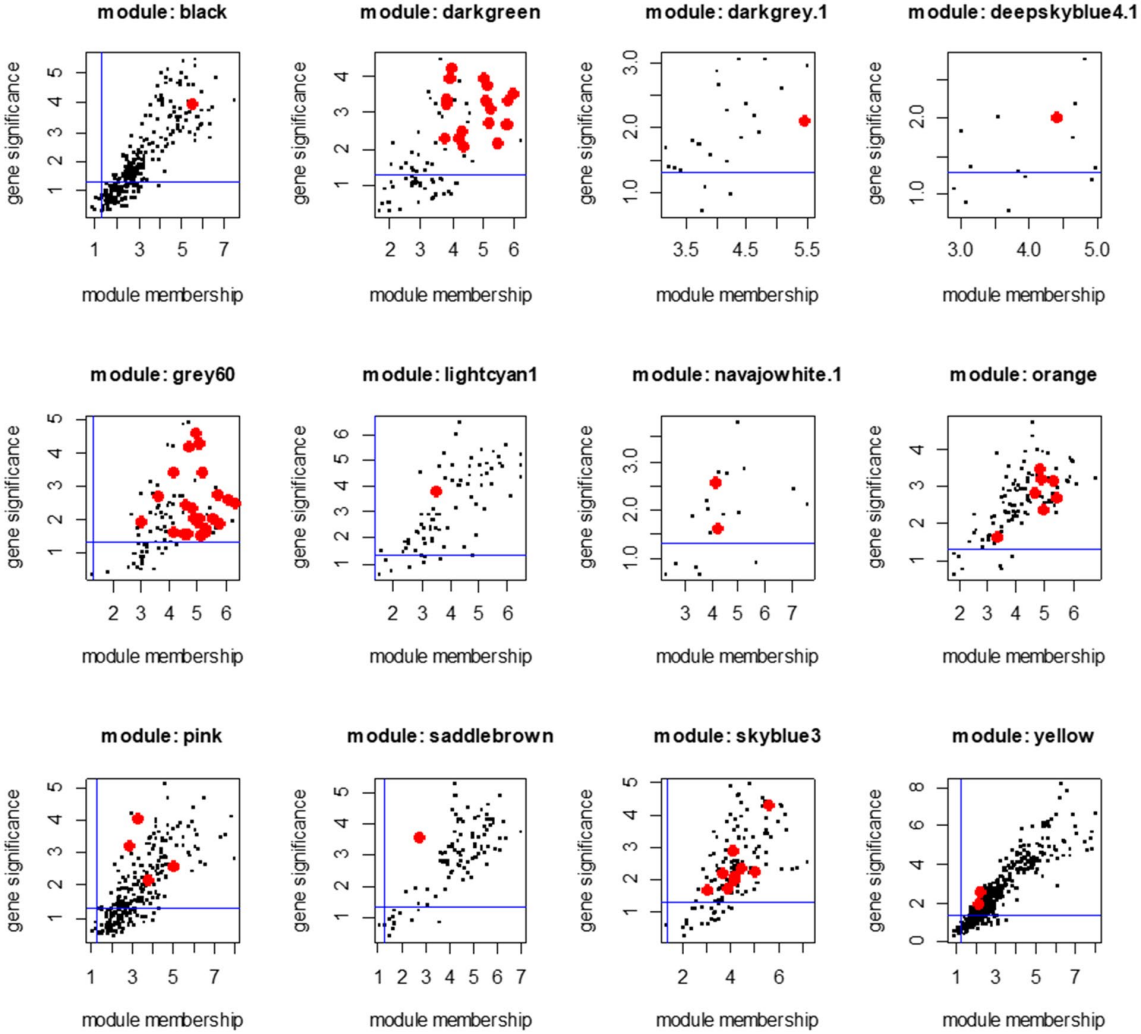
Hub miRNAs (red dots) identified in each co-expression module in C3H mice. The circulating miRNAs identified displayed strong memberships, based on WGCNA criteria, within each module and significant correlations with the HCC phenotype. Blue lines: p < 0.05.

Hub miRNAs were identified from the 16 HCC-associated modules by evaluating the intramodular membership and biological functions of each miRNA. The candidate miRNAs that showed good correlations with the module eigenvalues (FDR < 0.2) were considered as having strong memberships. The miRNAs in each module were also queried in the mirRDB functional miRNA database and then searched in TargetScan miRNA target database. We have identified 71 circulating miRNAs in 12 modules that demonstrated strong intramodular memberships and coexisted with their predicted gene targets in the same modules (Fig. 2).

#### Confirmation of HCC-associated circulating miRNAs in irradiated mice with heterogeneous genetic backgrounds

**Figure 3:**
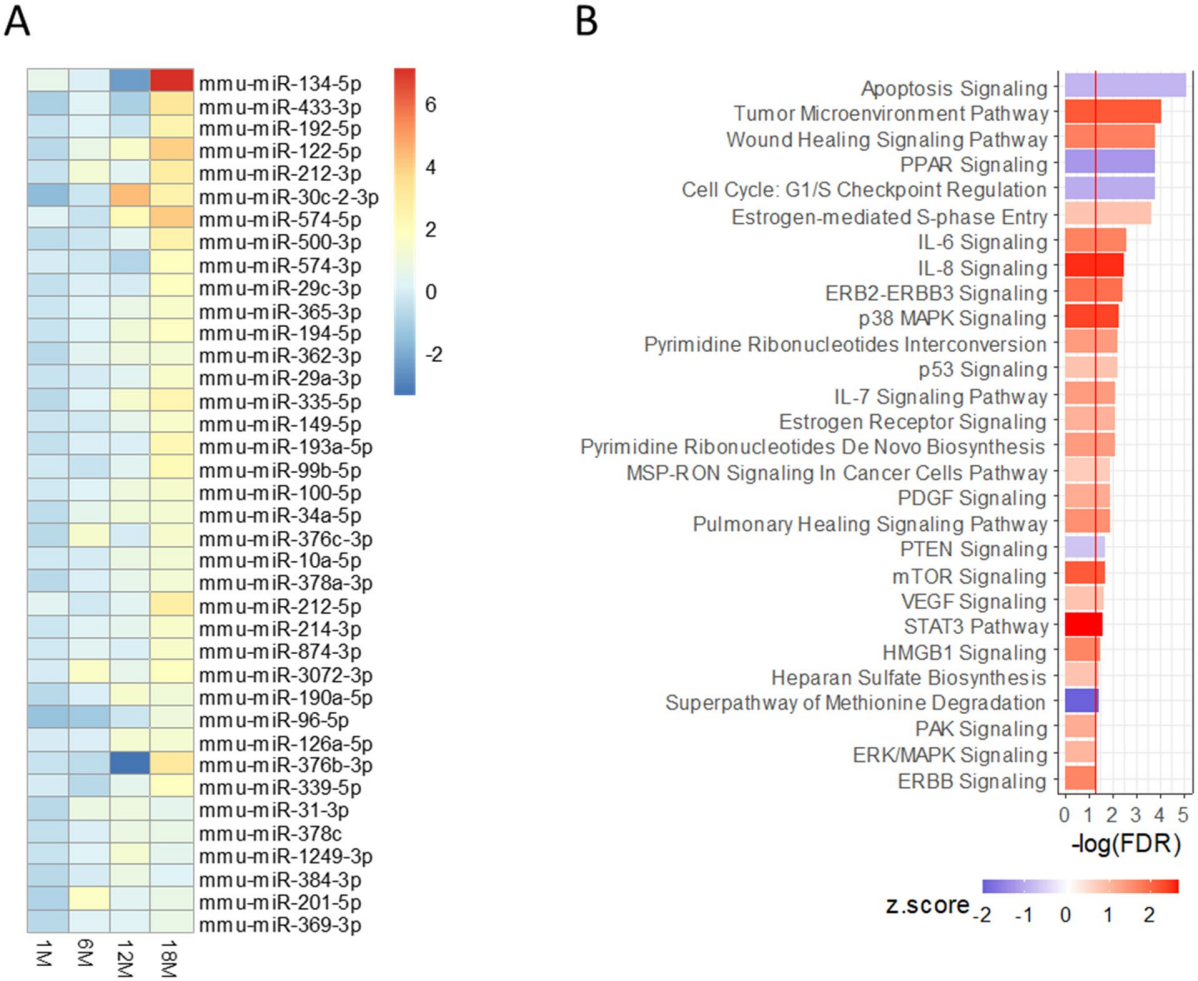
Validation of hub miRNAs in serially collected plasma samples from irradiated F2 mice diagnosed with HCC. (**A**) Normalized plasma levels of 38 circulating hub miRNA validated in F2 mice. (**B**) Signaling pathways of the predicated gene targets of the 38 validated circulating miRNA markers. The horizontal axis represents enrichment scores. The colors of the bars represent the z-scores of the pathways, red: activation; blue: suppression. The red line represents the cutoff of enrichment (FDR < 0.05).

An independent cohort of mice from the second-generation offspring (F2 mice) of C3H x BALB/c hybrids were irradiated and monitored for HCC. Plasma was collected from these mice 1, 6, 12 and 18 months after irradiation without the need for euthanasia. Of the 71 over-represented circulating hub miRNAs identified from tumor-bearing C3H mice, the plasma levels for 38 of those 71 miRNAs were also altered (FDR < 0.05) in HCC-bearing F2 mice at 18 months post radiation (Fig. 3A). Pathway analysis was performed by examining gene targets of these 38 miRNAs. Down-regulation of apoptosis signaling, cell cycle G1/S checkpoint, Pten signaling; upregulation of p38 Mapk signaling, p53 signaling, mTor, Vegf, Stat3 and Erk/Mapk signaling are amongst the significantly changed pathways affected by the 38 miRNA markers in HCC mice (Fig. 3B).

### Comparison of mouse and human HCC-associated circulating miRNAs

**Figure 4:**
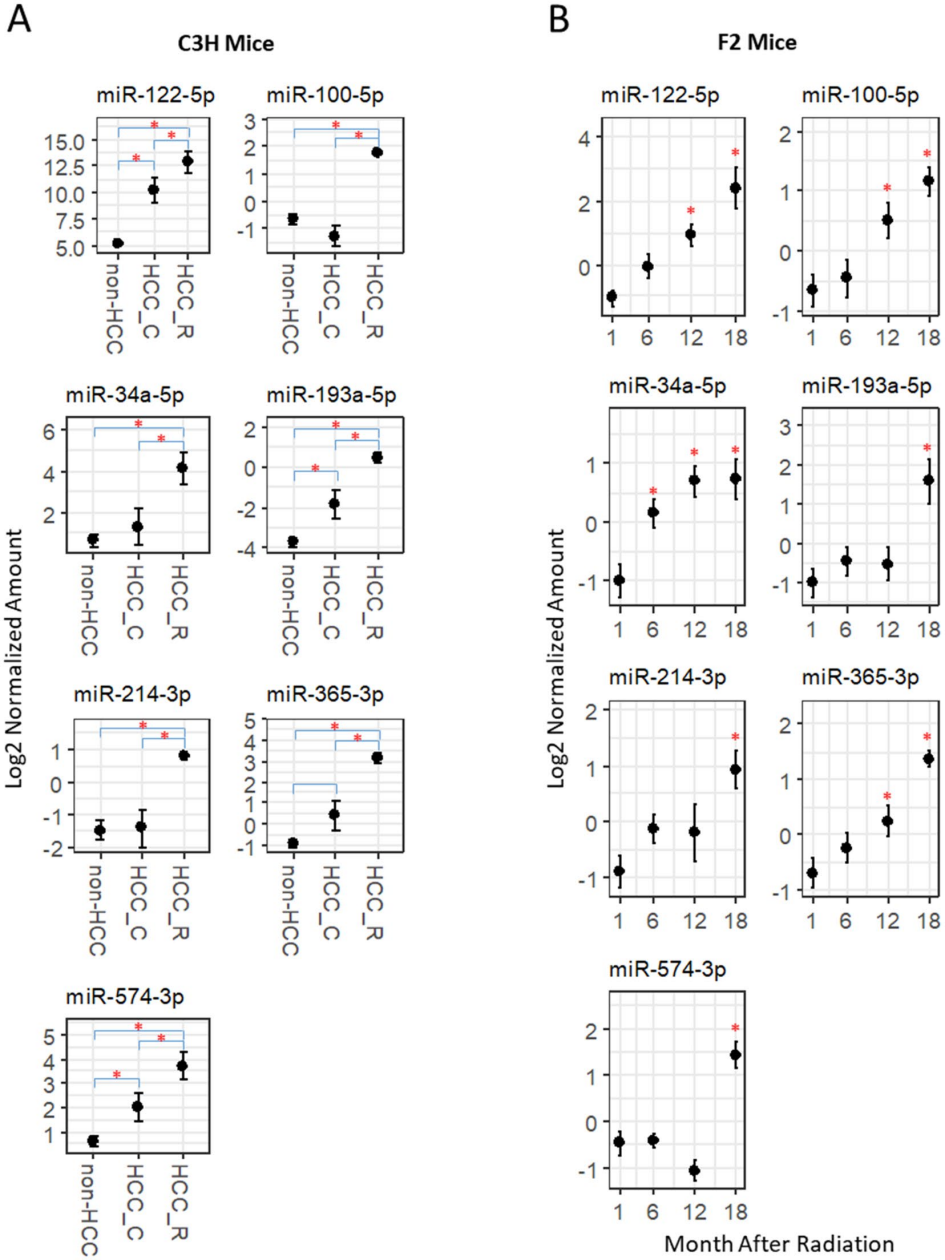
Human HCC-associated circulating miRNAs overlapped with mouse hub miRNAs in two strains of mice. (**A**) Mouse orthologs of human HCC circulating miRNA markers in the plasma C3H mice with HCC diagnosis. *HCC_R* radiation-induced HCC, *HCC_C* spontaneous HCC. Red star: significantly different between groups (p < 0.05). Error bar: standard error. (**B**) Mouse orthologs of human HCC circulating miRNA markers in the plasma of F2 mice with HCC diagnosis. Red star: significantly different from 1-month group (p < 0.05). Error bar: standard error.

We hypothesized that the functionally important hub miRNAs are conserved across different species and they are more likely the common biomarkers for HCC between mice and humans. Human HCC circulating miRNA markers were selected from a previously published study in which 12 circulating miRNAs were found significantly increased in 3 independent cohorts of HCC patients^17^. Among the 12, two miRNAs were species-specific and do not exist in mice. Seven of the remaining 10 miRNAs overlapped with the hub miRNAs in mice. The seven common miRNAs were significantly increased (p < 0.05) in C3H mice bearing radiation-induced HCC (Fig. 4A) and in F2 HCC-bearing mice at 18 months post radiation (Fig. 4B). In C3H mice, the seven miRNAs in radiation-induced HCC also showed significant increase when compared to spontaneous HCC (p < 0.05). In F2 mice, four of the 7 miRNAs, miR-122-5p, miR-100-5p, miR-34a-5p and miR-365-3p, increased significantly as early as 12 months after radiation (p < 0.05). MiR-34a-5p increased significantly at 6 months (p < 0.05). Of the 3 human HCC markers did not overlap with mouse hub miRNAs, miR-148a-3p and miR-224-5p were significantly increased in two mouse cohorts; miR-125b-5p only increased in C3H cohort.

### Early detection of HCC-associated circulating miRNA markers using change-point analysis

**Figure 5:**
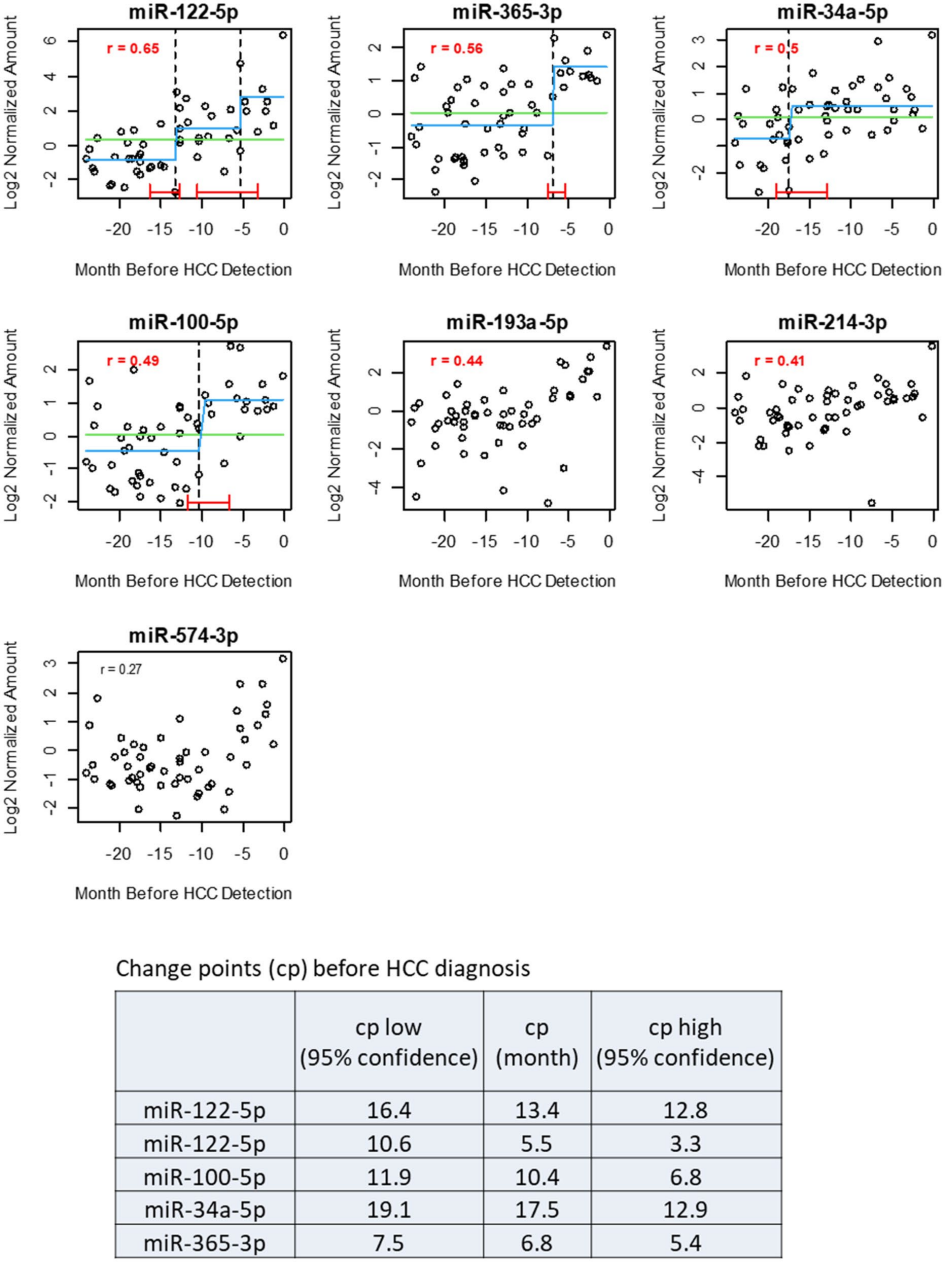
Change point analysis using the time-series data from the mouse orthologs of the 4 human circulating miRNA markers. Plasma levels for the circulating miRNAs in the F2 mice were plotted against the number of days counted backwards from HCC detection. r: correlation coefficient between y and x axes. Dash line: the identified change points with statistical significance (p < 0.05). Red line: 95% confidence interval for each change point detection. Blue line: fitted line of the regression model. Green line: fitted line of the null model.

In the F2 cohort, HCC was detected by necropsy at a range of 3 to 400 days after the last plasma collection. The plasma levels of all the seven human/mouse HCC-associated miRNAs were correlated with the number of days between plasma collection and the HCC detection. The highest correlations were from the 4 miRNAs that were elevated early, that is, at 12 months post-radiation (Fig. 5). The correlation of plasma miRNA levels as a function of time prior to HCC detection suggested direct associations of the 7 circulating miRNA markers with HCC progression.

To estimate how early the increased circulating miRNA markers were detectable before HCC diagnosis, we fitted the time series data for the circulating miRNAs in F2 HCC mice to a segmented linear regression model (strucchange::breakpoints)^26^ that detected change points in the regression data structures. The model identified significant change points in 4 circulating miRNAs (p < 0.05) among the 7 orthologs of the human HCC miRNAs. Two change points were identified for miR-122-5p at 13.3 months and 5.5 months prior to HCC detection. Change points were detected at 10.4 months for miR-100-5p, at 17.5 months for miR-34a-5p and at 6.8 months for miR-365-3p. The 95% confidence ranges at each change-point were also calculated (Fig. 5). The model did not detect any change points with statistical significances for the other 5 circulating miRNAs.

## Discussion

Early detection of HCC is critical for improvements in therapeutic outcomes. Currently, the only clinically used serological HCC marker is alpha-fetoprotein (AFP)^27–29^. And although it has been shown that clinical practice may benefit from the use of AFP, the marker suffers from low sensitivity and specificity for detecting early stage disease^30,31^. Previous studies suggested that circulating miRNAs were more sensitive than AFP and other markers, particularly when it came to detection of early-stage tumors^32^. These findings suggested that circulating miRNAs could serve as sentinels of disease onset in high-risk populations.

Conventional differential expression analysis, which usually identifies hundreds of differentially expressed miRNAs in tumor, generates significant species-specific bias. We implemented WGCNA analysis and identified a small number of hub miRNAs from a large pool of integrated miRNA-mRNA datasets. The selection for hub miRNAs was based on both mathematical and biological criteria. For example, the hub miRNAs had to be key members in a gene network that was significantly associated with the phenotype, i.e., HCC, and they also co-existed with their gene targets in the same network. The hub miRNAs were independently validated on a different genetic background. The validated HCC markers consisted of 38 circulating miRNAs that were involved in molecular pathways such as p53, apoptosis signaling, Pten signaling, mTor and Erk/Mapk signaling pathways. These pathways are known to be associated with HCC in both mouse models and humans^25,33,34^.

Many variables affect the results of circulating miRNA experiments and previous reports have revealed inconsistencies amongst data generated from different labs. We avoided common experimental artifacts by removing platelets during plasma separation and screening for hemolysis in plasma samples. We have also identified that there were significant fluctuations of circulating miRNAs levels at different ages (Supplementary Fig. S1). We therefore performed all the time-series analysis using age-corrected values. We selected the 10 human HCC circulating miRNAs markers because they were the only published miRNA panel that was validated in 3 independent patient cohorts^17^. Seven of the 10 human circulating miRNAs overlapped with the HCC-associated mouse hub miRNAs, suggesting the feasibility of using mouse models to identify human HCC markers.

In the C3H mouse cohort, the levels of circulating miRNA biomarker candidates were higher in radiation-induced HCC than in spontaneously arising HCC. C3H mice have a higher background HCC incidence, ~ 15%, compared to other inbred strains^35^. This suggests that within an HCC-susceptible genetic background, circulating miRNAs were more specific to carcinogen-induced HCC than for spontaneous HCC. In the time-series study using F2 mice, four circulating miRNAs, miR-122-5p, miR-100-5p, miR-34a-50 and miR-365-3p, showed significant correlations with the time remaining until tumor detection, suggesting their potential as tumor stage markers. These 4 miRNAs also have important biological functions in HCC and other cancers. MiR-122-5p is the most abundantly expressed miRNA in the liver, and its expression was reported to be downregulated in HCC tumors and elevated in the plasma of HCC patients^17,36,37^. Mir-122 is involved in cell cycle, p53 and other cancer signaling pathways^38,39^. MiR-34a-5p is directly regulated by the tumor suppressor p53 and has been reported to be dysregulated in various human cancers^39,40^. MiR-100-5p and miR-365-3p are known to modulate apoptosis and cell growth and both are downregulated in HCC tissues^41,42^. Low expression of miR-100-5p is associated with a worse prognosis for HCC^43^.

To estimate how early the changes occurred prior to HCC detection, we performed change-point analysis using the time-series data for the 4 sensitive circulating miRNA markers. We then used the breakpoint detection algorithm implemented in the strucchange R package designed for detecting changes within linear regression models^26^. The analysis suggested that the changes in the levels of these circulating miRNAs occurred months before HCC was detected, with some of the markers changed more than a year before the final diagnosis.

The early changes detected with the circulating miRNA markers suggested they can be used as sentinels for detection of early or even preneoplastic stage of HCC. Previous reports showed that circulating miR-122 and miR-100 were good markers not only for HCC, but also hepatitis and cirrhosis; miR-34a can differentiate HCC and cirrhosis from hepatitis; and miR-365-3p was specifically associated with HCC^17^. These results suggested that the combined usage of the 4 circulating markers can be useful in screening of HCC in certain high-risk individuals with chronic liver diseases. Other susceptible populations that could benefit from early HCC screening include individuals subject to environmental radiation exposures, e.g., astronauts in long-term deep space missions. For example, Edmondson et al., found HCC to be the second most abundant solid cancer induced by the types of radiations found in deep space^44^. These circulating miRNA sentinels have good sensitivity for detecting preneoplastic lesions or early-stage cancers. They have the potential to be valuable tools for health monitoring during future human space expeditions and facilitate the development of cancer preventative measures.

## Methods

### Mice and irradiation

The study was approved by the Institutional Animal Care and Use Committee at the University of Texas Medical Branch, Colorado State University and Brookhaven National Laboratory. All experiments were performed in accordance with relevant guidelines and regulations. The study was reported in accordance with ARRIVE guidelines. Male C3H mice were irradiated and maintained as described previously^25,35^. Blood samples were obtained via cardiac puncture at the time of necropsy. In total, 5 non-HCC and 8 HCC samples from C3H mice, including 5 spontaneous and 3 radiation-induced, were used in this study. The F2 mice were the second generation progeny from mating of BALB/c females and C3H males. Radiation and maintenance of F2 mice were described previously^45^. Blood samples of F2 mice of both sexes were collected from the tail vein at 1 month, 6 months, 12 months and 18 months after irradiation without sacrificing the animals. In total, 53 blood samples from 15 HCC-carrying F2 mice (7 females, 8 males) were used in this study. All HCC diagnoses were determined by histological examination from tissue biopsies.

### Plasma preparation

Blood was collected in tubes containing EDTA to prevent coagulation and was centrifuged at 1300 rcf for 10 min at 4 °C, followed by collection of plasma from above the buffy coat. The plasma was centrifuged at 1500 rcf for 10 min, and at 10,000 rcf for 20 min, at 4 °C. Each plasma sample was screened for hemolysis by measuring the hemoglobin concentration using the Harboe Direct spectrophotometric method with Allen correction^46^. Samples with Hb concentration > 0.02 mg/l were excluded from the study.

### RNA isolation and next generation sequencing

Total RNA from was isolated using 50 µl of mouse plasma. The sample volumes were brought up to 200 µl with water and then processed for RNA purification using the miRNeasy Serum/Plasma Advanced Kit from Qiagen. Synthetic miRNA mimics, UniSP2, UniSP4 were added to each sample before isolation. A QIAseq miRNA Library Kit was used to build molecular-barcoding-enabled sequencing libraries. Small RNA sequencing was performed at the Qiagen Genomics Service Core using an Illumina NextSeq500 sequencer. The read length was 76 nt, single-end read.

### Preprocessing of sequencing data

The sequence reads were aligned with *Mus musculus* reference genome GRCm38. MiRNAs were annotated according to mirbase_20. The aligned reads were de-barcoded to produce counts of Unique Molecular Index (UMI) corresponding to miRNAs. The UMI counts of miRNAs were normalized using the mean UMI counts of UniSP2 and UniSP4 spike-ins from each sample. The data were log2 transformed and used for subsequent analysis. Due to the significant fluctuation in circulating miRNA levels at different ages (Supplementary Fig. S1), we also normalized each miRNA with the values from HCC-free mice at the corresponding age.

### Identification of hub miRNAs in miRNA/mRNA co-expression networks

Construction of co-expression networks was performed using the WGCNA package in R according to the guidelines published by the developer. The gene expression data of the C3H cohort was published previously^25^ and deposited in GEO (GSE166653). We combined the gene expression profiles with miRNA sequencing data from each mouse. We subsequently removed miRNAs/mRNAs with low variances (coefficient of variance < 0.1). The resultant 30,439 features were used for network construction. The soft-threshold power was set at 6. The association of each co-expressed network/module with the HCC phenotype was analyzed using the limma package^47^ by fitting the eigenvalues of the networks against sample groups (normal, spontaneous HCC and radiation-induced HCC). False discovery rates (FDR) were calculated from p-values of F-tests. The significance cutoff was an FDR < 0.2. MiRNAs that displayed a strong membership (FDR < 0.2) with the HCC-associated modules were selected as hub-miRNA candidates. These candidates were screened for gene targets using TargetScan and mirRDB database and the miRNAs that co-existed with their predicted gene targets were identified as HCC-associated hub miRNAs.

### Confirmation of hub miRNAs and comparison with human HCC-associated circulating miRNAs

The R package limma was used to identify miRNA changes with statistical significance in the time series dataset (FDR < 0.05). Pathway analyses of the miRNA targets were performed using the Ingenuity Pathway Analysis software from Qiagen. The signaling pathways were considered significantly enriched with an FDR < 0.05 and absolute z-score > 0.5. Mouse orthologs of human miRNAs were retrieved from mirbase20. Correlation coefficients were calculated by spearman correlation analysis using normalized miRNA values and the negative number of days. Change points analysis was performed using the breakpoints detection algorithm from the strucchange package in R^26^.

## Supplementary Information

Supplementary Figure S1.

## Data Availability

Sequencing data of circulating miRNA profiles from C3H and F2 mice were deposited in Gene Expression Omnibus (GEO) and can be accessed via the accession number GSE196193.
