# Supplementary material for: Orthologs of human circulating miRNAs associated with hepatocellular carcinoma are elevated in mouse plasma months before tumour detection
Source: Sci Rep. 2022 Jun 28;12:10927. doi: 10.1038/s41598-022-15061-5 (PMC9240017; doi:10.1038/s41598-022-15061-5)
Supplement: Supplementary file 1 — Supplementary Figure S1. [file 41598_2022_15061_MOESM1_ESM.pdf]

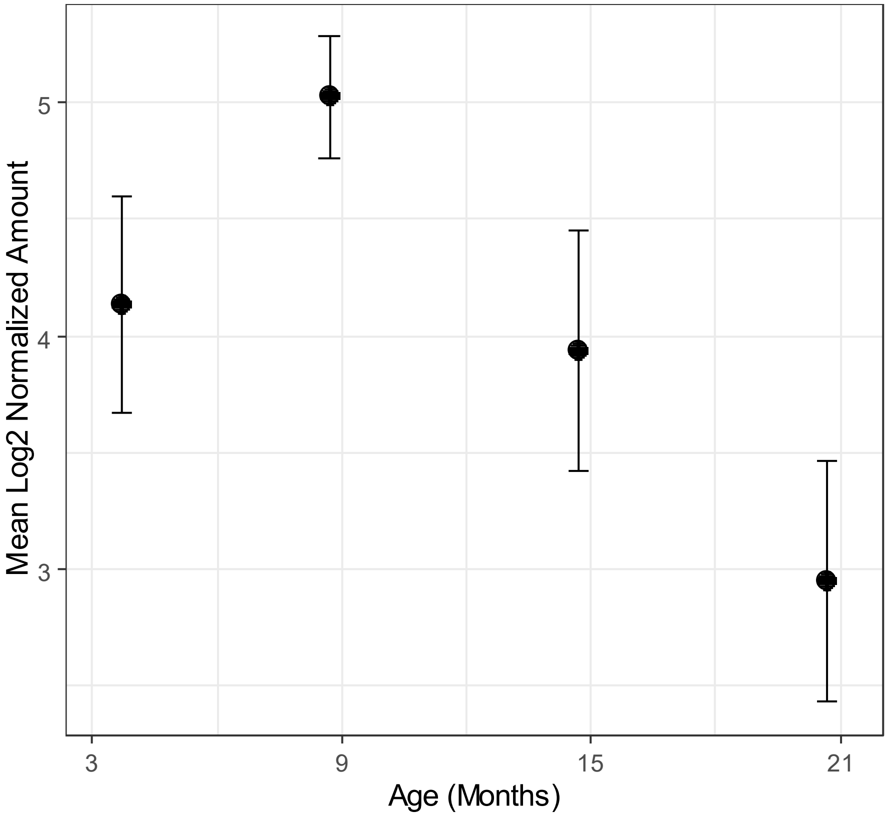

Fig. S1. Fluctuation of averaged hub miRNA levels in the plasma of F2 mice at different ages. Error bar: standard error.
